# Clinicians’ Involvement of Patients in Decision Making. A Video Based Comparison of Their Behavior in Public vs. Private Practice

**DOI:** 10.1371/journal.pone.0058085

**Published:** 2013-03-05

**Authors:** Nicole Mongilardi, Víctor Montori, Alejandro Riveros, Antonio Bernabé-Ortiz, Javier Loza, Germán Málaga

**Affiliations:** 1 Unidad de Conocimiento y Evidencia (CONEVID), Department of Medicine, Universidad Peruana Cayetano Heredia, Lima, Peru; 2 Department of Medicine, School of Medicine, Universidad Peruana Cayetano Heredia, Lima, Peru; 3 Knowledge and Encounter Research Unit, Division of Endocrinology and Internal Medicine, Mayo Clinic College of Medicine, Rochester, Minnesota, United States of America; 4 Epidemiology Unit, School of Public Health and Administration, Universidad Peruana Cayetano Heredia, Lima, Peru; 5 Cronicas Center of Excellence in Chronic Diseases, Universidad Peruana Cayetano Heredia, Lima, Peru; Tehran University of Medical Sciences, Islamic Republic of Iran

## Abstract

**Background:**

Little is known about the extent to which Peruvian physicians seek to involve patients in shared decision making, or about the variation in these efforts across different settings.

**Objective:**

To measure the extent to which Peruvian clinicians involve their patients in decision making and to explore the differences between clinicians’ behavior in private vs. public practice.

**Design:**

Videographic analysis.

**Participants and Setting:**

Seven academic physicians who provided care to patients in a public and a private setting participate in this study. All the encounters in both settings were filmed on one random day of February 2012. Approach: Two raters, working independently and in duplicate used the 12-item OPTION scale to quantify the extent of physician effort to involve patients in shared decision making (with 0 indicating no effort and 100 maximum possible effort) in 58 video recordings of usual clinical encounters in private and public practice.

**Results:**

The mean OPTION score was 14.3 (SD 7.0). Although the OPTION score in the private setting (mean 16.5, SD 7.3) was higher than in the public setting (mean 12.3 SD 6.1) this difference was not statistically significant (p = .09).

**Conclusion:**

Peruvian academic physicians in this convenience sample barely sought to involve their patients in shared decision making. Additional studies are required to confirm these results which suggest that patient-centered care remains an unfulfilled promise and a source of inequity within and across the private and the public sectors in Peru.

## Background

Traditionally, physicians made all the decisions without patient participation. Involving patients in decision making means respecting their autonomy [1], [2]. Furthermore, some evidence suggests that patient involvement in decision making decreases decisional conflict, increases patients` general satisfaction, improves knowledge, and improves short-term adherence [3], [4], [5], [6]. For these reasons, patient participation has been expanding, particularly in some healthcare systems in the Northern Hemisphere. Little evidence exists about the nature of patient involvement in decision making in emerging healthcare systems in the Southern Hemisphere where there is an increasing need to explore and understand the decision making process.

In Peru, clinicians’ attitudes and skills to involve patients in decision making are unknown, as is their actual practice of involving patients. Being Peru a middle-income country, there is a large gap between patients’ and doctors’ education, literacy, knowledge, and power. Consequently, many doctors may feel the need to take a paternalistic decision-making approach. This may be particularly pertinent in the public healthcare system caring for the most underserved. The public system is also noisier and busier [7] which may further reduce the likelihood of shared decision making. Thus, it is possible that differences in the clinicians’ involvement of patients exist, not only because of different patients’ characteristics, but also because of the setting?

In this study, we sought to determine the extent to which clinicians involve their patients in decision making, and whether there are differences in clinicians’ behavior seeing patients in private vs. public contexts.

## Methods

### Participants

We sought to recruit academic physicians who provide outpatient internal medicine and subspecialty care to patients with chronic conditions both in one public clinic and one private for profit clinic in an urban area in Lima. In these patients, successful care requires patient self-management, and the best course of action is often uncertain and sensitive to context and preferences, both conditions demanding patients play a more active role [8].

Patients were selected in a random day in February 2012, according to the order of arrival in the waiting room. We excluded children, patients with major learning barriers such as a hearing impairment, cognitive deficiencies or a language barrier, and patients seen with students.

Participants were naïve to the concepts we were measuring and had received no formal training in shared decision-making techniques. Physicians were asked to perform out-patient consultations as usual and were told that the videorecordings would be used to study certain aspects of physician-patient relationship. No other suggestions or information was given.

### Ethics Statement

All participants gave written informed consent and the study was approved by the “Universidad Peruana Cayetano Heredia” (code number:57146) and the “Hospital Nacional Cayetano Heredia” (code number: 07010) ethics committee.

### Approach

To determine the extent to which clinicians sought to engage patients in making decisions, we video recorded all the consultations the physician conducted with consecutive patients in the public and private clinics. To quantify this effort we used the OPTION scale (Observing PaTient InvOlvemeNt). This tool quantifies the extent to which physicians involve their patients in the decision making process, across twelve items used to rate consultations, either by video recordings, audio recordings and/or consultation transcripts. This tool has been used repeatedly with good validity and reliability [9], [10], [11], and has been translated to Spanish by the original developers [12]. Two Peruvian investigators (authors NM and GM) reviewed the items to ensure accurate representation of the original items in Spanish in Peru. The scale contains 12 items that are rated in a five point scale; 0 corresponding to “behavior not observed”, to 4 points which correspond to “behavior is exhibited to a very high standard”. Scores range from 0 to 48 points, and are transformed into a score of 0 to 100. Two investigators were trained and calibrated with the OPTION scale manual and seven standardized consultations. To evaluate the inter-rater reliability, a random sample of 20 consultations were rated independently by both raters; since we established satisfactory reliability scores (weighted kappa 0.79, p<0.001) only one rater analyzed the remaining 39 consultations.

### Data Collection

To characterize the participants, we noted physicians’ age, sex and years in practice and patients’ age, sex and grade of formal instruction.

For each consultation, we registered the setting (public or private), type of consultation (new complaint, review of a previous complaint, or both), visit length, amount of noise and number of interruptions. We considered an interruption as a sound or action that disturbed the ongoing process of the consultation i.e. phone ringing. We rated the noise with a subjective scale, from 0 to 4, being 0 no other noise heard beside the encounter itself, 1, small amount of noise, 2, moderate amount of noise that interferes rarely with the consultation (i.e. participants have to repeat what they are saying), 3, moderate amount of noise that interferes occasionally with the consultation and 4, excessive amount of noise that interferes frequently with the consultation.

For each decision, we noted the index problem (the one in which the parties spent the greatest amount of time and attention), and the type of decision (therapeutic or diagnostic). We rated the consultation considering only the index problem.

### Statistical Analysis

Analyses were performed with the 11.0 version of STATA for Windows, taking into account clustering of observations within physicians and settings. Univariate analyses of association were tested using nonparametric tests at a significance level of 5%. We used linear regression analyses to estimate the association between the total OPTION scores and participant, encounter, and decision variables.

## Results

### Participant and Consultation Characteristics

Of the 14 physicians who met the inclusion criteria, seven were excluded; two because of their low number of consultations per week, two because they did not have a fix schedule in the private for profit clinic, one because students were always present in his public setting’s encounters and the last two because they refused to participate. All physicians had seven or more years of practice and had not received risk communication or shared decision making training (n = 7).

Of the patients approached, 59 (approximately 70%) agreed to participate and were enrolled in this study (30 at the public hospital and 29 at the private setting). One patient from the private setting was excluded from the analysis because the consultation length was 44 minutes (an extreme outlier). Thus, only 58 recordings were used. The number of consultations per physician was 8.29+- 1.80 (4.29+- 1.11 in the public setting and 4+- 1.15 in the private setting). The most common index problems were diabetes (12/58), rheumatoid arthritis (5/58) and anemia (4/58). Overall, 15 (26%) interviews were new; 41 (71%) were review and 2 (3%) were composite. 48 (83%) required a decision about therapy. The mean length of interviews was 13.2 minutes (SD 5.2). See details in **Table 1**.

**Table 1:** Characteristics of participants and consultations in the two study settings.

Physician characteristics	N = 7	
*N (%) female	2 (29)	
Mean age (SD)	47 (9)	
Years in practice (SD)	15 (10)	
**Patient characteristics**	**Public clinic(n = 30)**	**Private clinic(n = 28)**
*N (%) female	24(87)	23 (86)
Mean age (SD)	54 (13)	52 (19)
*N (%) illiterate, elementary education, high school.	21 (71)	15 (43)
*N (%) advanced degree	9 (29)	13 (57)
**Consultation characteristics**		–
Mean length, minutes (SD), range	12.1 (3.9), (6–28)	14.4 (6.2), (5–27)
*N (%) with no interruptions	7 (24)	15 (47)
*N (%) with 1 interruption	12 (45)	9 (36)
*N (%) with 2 or more interruptions	11 (31)	4 (18)
Noise (0–2 points out of 4)	53.8%	99%
Noise (3–4 points out of 4)	46.2%	1%

* N are absolute numbers, while % are ponderated by cluster.

### Interrater Reliability and Internal Consistency of the OPTION Scale

Chance-adjusted inter-rater reliability (weighted kappa) of each OPTION item varied between 0 and 0.7 and kappa for the overall OPTION scale was 0.79, consistent with substantial agreement. We obtained no value for item 10, because all scores were 0. Cronbach’s alpha coefficient of the overall OPTION scale was 0.81, indicating a good internal consistency with little redundancy in the scale.

### Physician Effort to Involve Patients (OPTION Score)

OPTION total scores ranged from 2.1 to 31.3, with a mean score of 14.3 (SD 7.0), obtaining 12.3 (SD 6.1) in the public setting and 16.5 (SD 7.3) in the private setting (p = 0.09) (in a range of 0–100). There was a statistically significant difference between each physicians’ efforts to involve patients in decision making (p = 0.02, ANOVA).

Items 1 (“The clinician draws attention to an identified problem as one that requires a decision-making process”) and 12 (“The clinician indicates the need to review the decision (or deferment)”) had the greatest scores with at least a mean score of 1 out of 4 (minimal attempt to exhibit the behavior). Item 10 (“The clinician elicits the patient’s preferred level of involvement in decision-making”) had a value of 0 in all encounters. Only 2 physicians obtained points in item 3 (“The clinician assesses the patient’s preferred approach to receiving information to assist decision-making”); each obtained 1 out of 4 points in 2 encounters. In item 6 (“The clinician explores the patient’s expectations about how the problems are to be managed”), all encounters in the public setting obtained a score of 0, compared to 18 (64%) of the private encounters. See details in **Table 2**.

**Table 2:** Mean option scores per item in the public and in the private setting.

	Publicsetting	Private setting	p	Total
**Item 1**	0.82/4	1.19/4	0.16	0.99/4
**Item 2**	0.43/4	0.62/4	0.79	0.52/4
**Item 3**	0.00/4	0.09/4	0.27	0.04/4
**Item 4**	0.33/4	0.33/4	0.63	0.33/4
**Item 5**	0.71/4	0.79/4	0.8	0.39/4
**Item 6**	0/4	0.39/4	<0.001	0.19/4
**Item 7**	0.49/4	0.66/4	0.43	0.57/4
**Item 8**	0.97/4	0.85/4	0.8	0.91/4
**Item 9**	0.47/4	0.73/4	0.07	0.59/4
**Item 10**	0/4	0/4	NA	0/4
**Item 11**	0.54/4	1.17/4	0.01	0.84/4
**Item 12**	1.14/4	1.07/4	0.88	1.11/4
**Total**	12.27/48	16.45/48	0.09	14.25/48

The OPTION total score was not associated with participant or encounter characteristics (data not shown), except for type of consultation which correlated significantly: new consultations (n = 15) had a mean score of 9.3 (SD 2.6) compared to review and composite consultations (n = 43), which had a mean score of 15.2 (SD 7.1) (p<0.001). Using linear regression analysis, the length of visits were not associated significantly with mean total OPTION score, although there was a trend toward higher OPTION scores with longer visits (p = 0.08). When this analysis was stratified by setting, the coefficient obtained from the public and the private setting were p = 0.98 and p = 0.07, respectively. In the multivariable model, each minute of increase in visit duration was associated with a statistically significant increase in the OPTION score of 0.36 (95%CI: 0.01–0.69). after adjusting for number of interruptions, patient’s education level, and setting.

## Discussion

### Our Findings

Physicians in both settings sought to involve patients to a similar extent, despite the differences in patients’ education, number of interruptions and noise between the private and the public setting. Yet, this extent reflected a very low level of patient involvement in the decision making process. Although the mean score in the private setting was somewhat higher than in the public setting, this difference was not significant. The observed trend of lesser patient involvement in the public setting could be due to its hostile environment for shared decision making (noisy, with constant interruptions and time constraints). Another reason could be because of physicians’ perception of patients not desiring to be involved in decision making, due to patients not actively participating in the encounter or to physicians’ preconceived notions because of patients’ different sociocultural backgrounds.

Private setting consultations show higher scores for specific items 6 and 9 (“The clinician explores the patient’s expectations (or ideas) about how the problem(s) are to be managed” and “The clinician offers the patient explicit opportunities to ask questions during decision making process”). This can probably be explained because patients in the private setting tended to participate more actively in the consultations than patients from the public setting. (i.e. ask more questions, reveal their concerns).

The only predictors of increased patient involvement were review encounters (in univariate analysis) and longer encounters (in multivariate analysis).

### Our Findings in the Context of the Literature

Our study shows one of the lowest levels of patient involvement reported in the literature [13], [14], [15]. This could reflect (a) bias of the assessors toward lower scores, (b) poor performance of the OPTION score in this setting, or (c) reduced effort to involve patients in this context by these clinicians. The latter could be due to lack of physician skills or disposition toward patient involvement, and limited expectations of patients to participate, even among those with higher education. Clinicians made no effort to determine patients’ preferred level of involvement, perhaps assuming that no involvement was desired.

We found a trend toward greater patient involvement in longer visits. This is consistent with the literature [13], [15], [16], [17]. The association was not stronger perhaps because study duration reflects more the structural problems of delivering care in the settings studied (e.g. interruptions), rather than longer visits because of efforts to engage patients.

### Limitations and Strengths

The most important limitations of our study relate to (a) small sample of clinicians, (b) the unknown representativeness of patients who opted to participate recognizing that 30% opted out of the study, (c) the convenient selection of clinics which may not be representative of other public or private clinics in Peru, (d) cross-sectional nature of the study which does not allow for causal inferences, and (e) the inability to blind the assessors to setting and physician, given that this is reflected in the videos. The OPTION scale has itself some limitations that affect its performance and interpretations. First, empirical evidence concerning its factorial structure is unclear; the majority of studies have found more than one factor. Second, inter-rater reliabilities may be overestimated because items are non-independent, and finally, most studies have not evaluated the validity of this scale. [18].

Our study also has some relative strengths. To our knowledge, this is the first study directly observing the patient engagement behaviors of Peruvian clinicians and using the OPTION score to assess such encounters. Also, our instrument was validated for its use in Peru, and used by trained and calibrated investigators, being this reflection of our results which show a good internal and external correlation.

### Conclusions

The Peruvian physicians studied involve patients in the decision making process to a very limited extent. This study offers preliminary evidence of a major deficiency in healthcare delivery, i.e., the lack of patient-centered encounters. As such, this study requires replication in a larger sample more representative of patients, clinicians, and settings. If confirmed, a large effort to train and tool clinicians and patients will be needed along with the identification and removal of barriers and the promotion of facilitators of shared decision making.
